# Large‐Scale Evaluation of Traditional Chinese Medicines Reveals Potential PPARγ Modulators for Type 2 Diabetes Management

**DOI:** 10.1002/jcla.70215

**Published:** 2026-04-01

**Authors:** Mingyuan Dou, Shandra Jones, Juan Montes, Jonathan Ritter, Li‐Yu Shih, Chia‐Jung Chan, Ying‐Chou Lin, Min Chen, Shih‐Yin Chen, Ning Wu

**Affiliations:** ^1^ Department of Mathematical Sciences, School of Natural Sciences and Mathematics The University of Texas at Dallas Dallas Texas USA; ^2^ Department of Biological Sciences, School of Arts and Sciences Southeastern Oklahoma State University Durant Oklahoma USA; ^3^ Graduate Institute of Integrated Medicine China Medical University Taichung Taiwan, Republic of China; ^4^ Genetic Center, Department of Medical Research China Medical University Hospital Taichung Taiwan, Republic of China; ^5^ Department of Accounting and Finance, John Massey School of Business Southeastern Oklahoma State University Durant Oklahoma USA; ^6^ School of Chinese Medicine China Medical University Taichung Taiwan, Republic of China

**Keywords:** drug effect, PPARγ gene, promoter activity, traditional Chinese medicine, transcription; type 2 diabetes

## Abstract

**Background:**

Peroxisome proliferator‐activated receptor‐gamma (PPARγ) is a pivotal nuclear receptor regulating glucose and lipid homeostasis, making it a primary therapeutic target for type 2 diabetes mellitus (T2DM). Despite the therapeutic potential of Traditional Chinese Medicine (TCM), a systematic, large‐scale evaluation of its modulatory effects on PPARγ remains insufficient.

**Methods:**

We developed a high‐throughput screening platform utilizing a pGL4.17‐PPARγ promoter‐luciferase reporter system in 293 T cells. A library of 639 TCM samples (345 single formulas, SF; 294 multiple formulas, MF) was evaluated. Leading candidates were validated through RT‐qPCR, Western blotting, and ELISA to assess PPARγ mRNA expression and protein abundance.

**Results:**

Screening identified 139 TCMs that enhanced PPARγ activity, with 47 SF and 2 MF achieving an effective rate (ER) exceeding 60%. Notably, 25 of these are novel candidates for T2D, while 24 align with previous reports. Specifically, Astragali Radix (S241) and Puerariae Thomsonii Radix (S258) were identified as potent inducers, significantly upregulating PPARγ at both transcriptional and translational levels. Conversely, 55 samples, including *Gymnema sylvestre* (S169) and Paris polyphylla (S3), exhibited robust inhibitory effects, reducing ER by −52% to −99%.

**Conclusions:**

This study identified 45 novel TCM candidates for T2D management. While several TCMs demonstrated PPARγ inhibition despite their traditional use for diabetic symptom alleviation, our findings suggest that a comprehensive evaluation is essential to balance their immediate clinical benefits with long‐term metabolic management and potential side effects.

## Graphical abstract

Identifying novel treatments for diabetic management is always a competitive research topic all over the world. PPAR‐γ is one of the key regulators involved in glucose and lipid metabolism and improves insulin sensitivity and glucose homeostasis. This study investigated the impacts of 639 traditional Chinese medicine (TCM) on PPAR‐γ gene promoter activity. The results identified 45 novel TCMs as the potential new candidates for T2D management. However, 55 TCMs that are used to alleviate T2D side effects currently may need reevaluation of their applications because they reduced PPAR‐γ gene expression and may negatively impact T2D management.

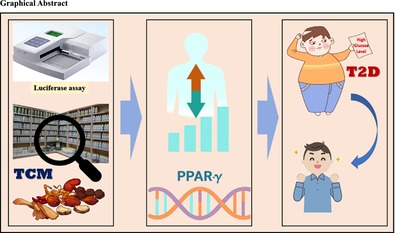

**Abbreviations**
ANOVA: analysis of variance
cRLU: calibrated RLU
DMEM: Dulbecco's Modified Eagle Medium
DMSO: dimethylsulfoxide
ER: effective rate
FABPs: fatty acid binding proteins
FDR: false discovery rate
GLUT4: glucose transporter 4
LPL: lipoprotein lipase
MF: multiple formulas
PPARγ: peroxisome proliferator‐activated receptor‐gamma
RLU: relative light unit
SD: standard deviation
SF: single formulas
T1D: Type 1 diabetes
T2D: Type 2 diabetes
TCM: traditional Chinese medicine

## 1 Introduction

Diabetes mellitus encompasses a widespread set of metabolic disorders primarily due to decreased insulin production, impaired glucose absorption, and increased glucose synthesis. Globally, around 415 million people have diabetes, with 193 million undiagnosed cases. The two main types of diabetes are type 1 diabetes (T1D), resulting from the autoimmune destruction of insulin‐producing beta cells in the pancreatic islets, and type 2 diabetes (T2D), caused by a combination of insulin resistance and relative insulin deficiency. In the U.S.A., nearly 10% of the population was affected by diabetes in 2023, with 90%–95% of reports being T2D and approximately 5300 new cases occurring each year in children, adolescents, and young adults, which is mainly attributed to the combination of genetic and environmental influences. The disruption in metabolism linked to diabetes leads to subsequent pathological alterations in several body organs, creating significant challenges for individuals with diabetes and the healthcare system. Unlike T1D, T2D is often associated with a combined disorder of insulin secretion and action and is marked by insulin resistance due to the deleterious effects of excess lipids on various organs, enhanced inflammatory signaling, and activation of stress pathways in the endoplasmic reticulum. Insulin resistance refers to a decreased sensitivity of tissues to insulin, which is typically marked by inefficient insulin‐stimulated glucose absorption in tissue cells and irregular regulation of liver glucose production by insulin. Currently, there are several classes of T2D medicines, each of which lowers blood sugar in a different way, including increasing insulin production of the pancreas, inhibiting the production and release of sugar from the liver, inhibiting intestinal absorption of glucose, reducing the reabsorption of sugar from the urine in the kidneys, decreasing the mobility of the stomach, and increasing the insulin sensitivity of the cells. However, due to the complex mechanisms of T2D that affect multiple organs, no single drug is completely effective in treating T2D, which suggests that future treatment development may need to address the root causes, including overeating, energy imbalances, and inflammatory responses.

Peroxisome proliferator‐activated receptor gamma (PPARγ), a receptor intimately involved in glucose and lipid metabolism, is essential in enhancing insulin sensitivity and reducing insulin resistance in body tissues. The PPARγ receptor activities are affected by posttranslational modifications, receptor turnover, polymorphisms, splice variants, and coactivators/corepressors [1]. PPARγ boosts the expression of glucose transporter 4 (GLUT4), which is crucial for glucose uptake in adipose and muscle tissues, thus directly enhancing insulin sensitivity [2]. In addition, PPARγ upregulates adiponectin, an adipokine known for improving insulin sensitivity and exhibiting anti‐inflammatory effects [3]. Further, PPARγ also influences the expression of fatty acid binding proteins (FABPs) and fatty acid translocase CD36, the keys in fatty acid transport and metabolism, thereby affecting lipid utilization and insulin sensitivity [4]. Lipoprotein lipase (LPL), an enzyme involved in the breakdown of triglycerides, is another target of PPARγ, which is essential for lipid management and insulin sensitivity [5]. PPARγ plays a key role in adipogenesis. By regulating genes involved in lipid storage and metabolism within fat cells, it ensures the proper functioning of adipose tissue, a key factor in maintaining insulin sensitivity, and maintains the balance in lipid and glucose metabolism, which is crucial for reducing the risk of insulin resistance and metabolic disorders like T2D. Treatments that increase PPARγ activity, such as thiazolidinediones, are currently used in T2D management, which act as PPARγ agonists, improving tissue insulin sensitivity and helping to prevent and treat hyperglycemia, hyperlipidemia, and insulin resistance [6].

Traditional Chinese medicines (TCM) have shown potential in T2D treatment, with some compounds being confirmed to act as PPARγ agonists, which mirrors the mechanism of thiazolidinediones to activate PPARγ gene, improve cell insulin sensitivity, and regulate blood sugar levels [1]. These results suggest that TCM may aid in regulating blood sugar levels and improving insulin sensitivity and open new avenues for integrated diabetes treatment approaches. Although there is strong evidence that some Chinese herbal medicines are effective in managing T2D, the underlying mechanisms remain unclear. However, any treatment that can increase insulin sensitivity will be of great benefit to T2D patients. As an alternative medicine, TCM has great potential in the treatment of T2D, not only because of its wide range of drug selection but also because multiple drugs can be combined for the same therapeutic purpose based on TCM theory. In addition, most Chinese herbal compound prescriptions use natural products as basic drug materials rather than the synthetic chemicals used in Western medicine, which makes the therapeutic effects of Chinese medicine more comprehensive than Western medicine, and the side effects are milder or even smaller.

To comprehensively explore the effectiveness of TCM in the T2D management, this study used human cell lines to explore the effects of different TCM formulars on the activity of the PPARγ gene promoter region. The study included 639 TCM formulars with 345 single formulas (SF) and 294 multiple formulas (MF). Luciferase assay was employed to inspect PPARγ gene promoter activity in human‐originated 293 T cells after the treatments of different TCM formulars. Through precise data processing and analysis, the effects of different TCM formulars on the activity of the PPARγ gene promoter were identified. The TCM formulars that enhanced the activity of the PPARγ gene promoter were identified as the potential candidates for T2D management, while the TCM formulars that reduced the activity of the PPARγ gene promoter were identified as the targets that should be avoided by T2D patients. The results of this study provided evidence for the strategy of T2D management using natural products and suggested the targets for future studies of the mechanisms and clinical applications.

## 2 Materials and Methods

### 2.1 Preparation of Traditional Chinese Medicines

A total of 639 TCM formulars including 345 SF and 294 MF obtained from the School of Chinese Medicine, Chinese Medical University, Taichung, Taiwan, ROC were included in this study with all TCM formulars names being standardized using Chinese Herbal Medicine Database (https://herbaltcm.sn.polyu.edu.hk/). All formulas were prepared following the standard TCM preparation procedures, dried, and then dissolved in Dimethylsulfoxide (DMSO) with a concentration of 200 μg/μL before allocating into 96‐well plate in 100 μL Gibco Dulbecco's Modified Eagle Medium (DMEM) (Thermo Fisher Scientific, Richardson, TX, USA) to the final concentration of 2 μg/μL for the following drug treatment tests.

### 2.2 PPARγ Promoter Plasmid DNA Construction

**FIGURE 1:**
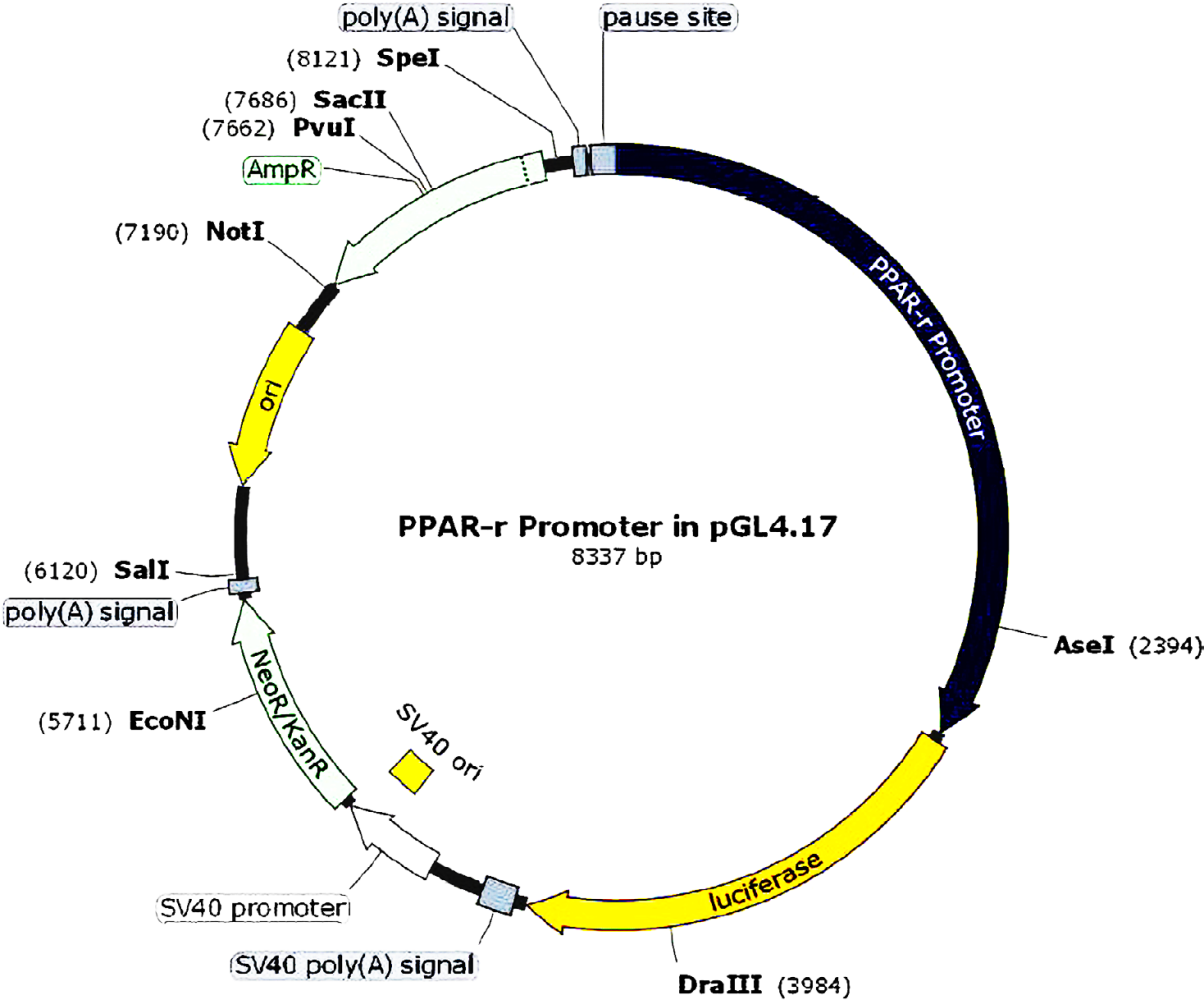
Constructed pGL4.17‐ PPARγ promoter plasmid.

The human PPARγ promoter sequence (2776 bp) was retrieved from GenBank core nucleotide BLAST database (Accession ID: NG_011749) and was subcloned into pGL4.17[*luc2*/Neo] vector (Promega, Madison, WI, USA) with both 5′ filling sequence (GGC CTA ACT GGC C) and 3′ filling sequence (GGC CTC GGC GGC C), respectively, through the customized plasmid DNA construction service provided by AllBio Science Inc. (Taichung, Taiwan, ROC). The resulted pGL4.17‐PPARγ promoter plasmid with 2803 bp insert was ready for transfection experiment (Figure 1).

### 2.3 TCM Preparation and Selection

A total of 639 standardized TCM formulas, comprising 345 single‐herb and 294 compound formulas, were sourced from a GMP‐certified pharmaceutical facility. To determine the most effective solvent for the powdered formulas, a solubility screening was conducted using water, ethanol, a 1:1 (v/v) water‐ethanol mixture, and DMSO. DMSO was selected as the optimal solvent for all subsequent experiments. Following dissolution, the samples were centrifuged to remove insoluble debris, and the supernatants were utilized for drug screening. Based on cell viability assays (shown in Supporting Information), a final concentration of 2 μg/μL was employed to ensure maximal efficacy without compromising cell viability.

### 2.4 Transfection of PPARγ Gene Promotor Region Into Cells

After seeding 1 × 10^6^ 293 T cells (ATCC, Manassas, Virginia, USA) in a 10 cm petri dish with 10 mL of Gibco DMEM (Thermo Fisher Scientific, Richardson, TX, USA) at 37°C with 5% CO_2_ overnight, the preconstructed pGL4.17‐PPARγ promoter plasmid was transfected into the 293 T cell line. Briefly, 5 μL of 1 μg/μL pGL4.17 plasmid and 15 μL of HyFect DNA Transfection reagent (LEADGENE, Tainan, Taiwan, ROC) were mixed in 300 μL of serum‐free media for 5 min and then tipped gently for another 25 min before transferring to 6 mL of 293 T cell culture and incubating at 37°C with 5% CO_2_ overnight. After transfection, 10 μL of 500 μg/mL Geneticin Selective Antibiotic (G418 Sulfate) was applied to every 10 mL of culture for transfected cell selection. The transfected cells were subcultured in a 96 well plate with 1.5 × 10^4^ cells in 100 μL media per well and incubated at 37°C with 5% CO_2_ overnight. The transfected cells were ready for drug screening.

### 2.5 TCM Treatments

The transfected cells were cocultured with 2% of each SF or MF at 37°C with 5% CO_2_ for 5 h with cultural medium plus empty pGL4.17 [luc2/Neo] vector as negative control and 1% DMSO plus pGL4.17‐PPARγ promoter plasmid as positive control. After treatment, Luciferase assay was performed using The Luciferase Assay System (Promega, Madison, WI, USA) following manufacturer's instructions. The absorbance of luminescence was measured using Mithras LB 940 Multimode Microplate Reader (Berthold Technologies, Bad Wildbad, Germany). The relative light unit (RLU) of each sample was recorded.

### 2.6 Data Processing

All resulted SF and MF original RLU data were calibrated by subtracting the positive control RLU (RLU_positive control_) in each experimental group to obtain calibrated RLU (cRLU) as below [1].

$$\text{cRLU}={\mathrm{RLU}}_{\text{sample}}-{\mathrm{RLU}}_{\text{positive control}}$$

The effective rate (ER) was defined as the percentage of increase in the activity of the PPARγ promoter region by each TCM formula treatment and was determined by dividing the RLU_positive control_ from the cRLU of each sample as shown below [2].

$$\text{Effective ratio}\;\left(\mathrm{ER}\right)\;\left(\%\right)=\left(\text{cRLU}/{\mathrm{RLU}}_{\text{positive control}}\right)\times 100\%$$

The effects of TCM on the activity of PPARγ promoter region may have three potentials including enhancement, no effect, and reduction. Therefore, the data obtained in this study were divided into enhancement, ineffective, and reduction groups after a series of statistical processing steps. Briefly, all resulted ER values were arranged in order from the largest positive to the smallest negative with both SF and MF data mixed as one dataset to calculate the mean value (*μ*) and standard deviation (SD) (*σ*). The Z score of each sample was then determined as follows [3].

$$Z\;\text{score}=\left(\mathrm{ER}-\mu \right)/\sigma$$

**FIGURE 2:**
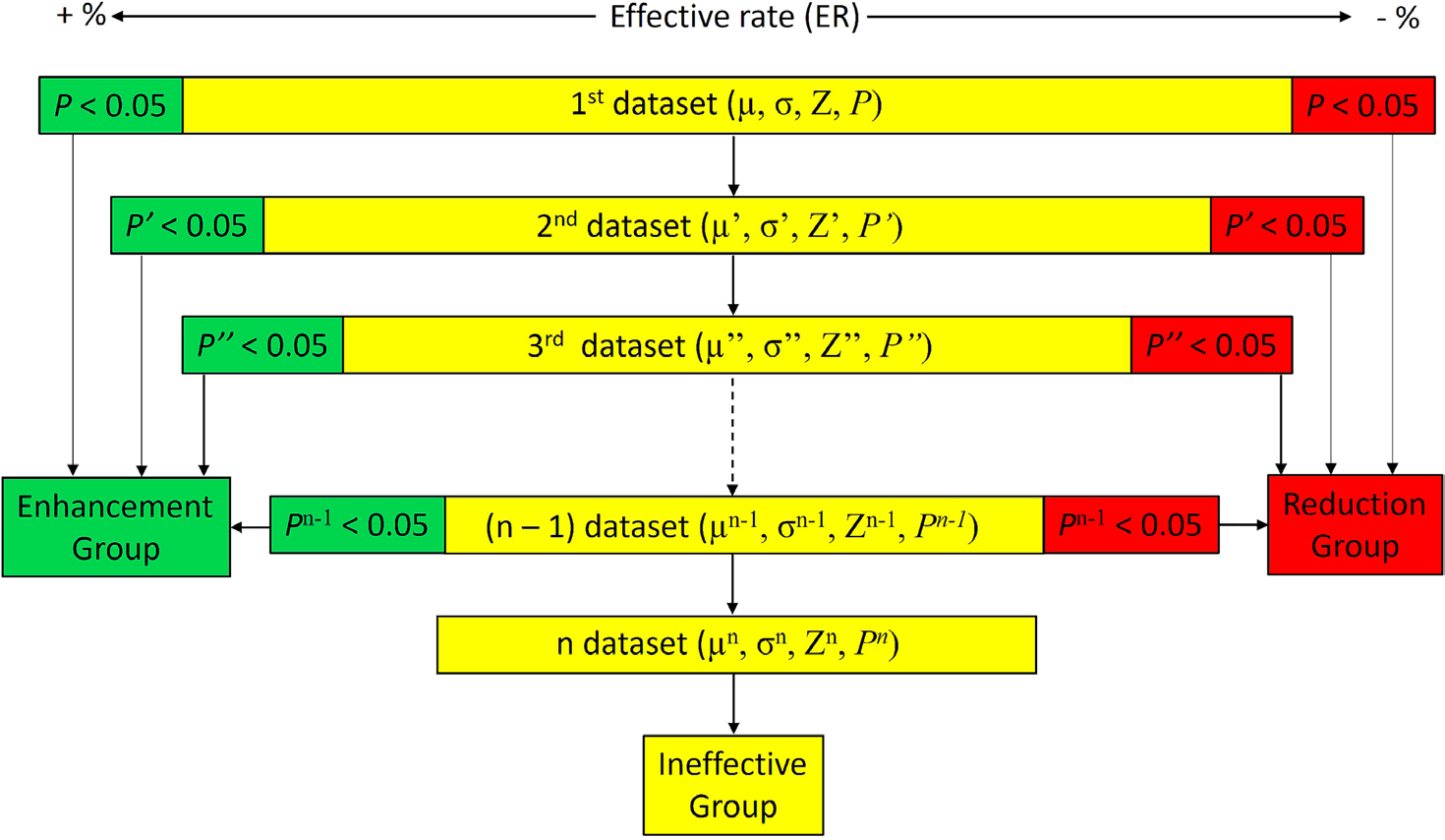
Grouping flow chart for 639 TCM formulars based on sample *p* value.

The *p* value of each sample was calculated using Microsoft Excel (Microsoft, Redmond, WA, USA) with the following equation [4].

$$p\;\text{value}=2\times \mathrm{MIN}\left(\text{NORM}.S.\text{DIST}\left(\mathrm{ABS}\left(Z\right),\text{TRUE}\right),1–\text{NORM}.S.\text{DIST}\left(\mathrm{ABS}\left(Z\right),\text{TRUE}\right)\right)$$

where *Z* was the standardized *Z* score obtained from equation [3]. After assigning a *p* value to each sample in the dataset, samples with a *p* value < 0.05 were removed from the dataset and relocated them to either the enhancement or reduction groups based on their ER values. The remaining data formed the second dataset where the new mean (*μ*′), SD (*σ*′), *Z* scores (*Z*′), and *p* values (*P*′) were calculated again, and the samples with their *P*′ value less than 0.05 were moved to either enhancement or reduction groups according to their ER values. The same steps were repeated until there was no sample's *p* value < 0.05 in the newly formed dataset. The last dataset was assigned as ineffective group (Figure 2). After the finalization of all data into three groups, the last set of *μ*, *σ*, *Z* scores, and *p* values were calculated for each group, and then, based on the *p* values, the false discovery rate (FDR) (*Q* value) of 5% was calculated using equation to validate the confidence of the sample data in each group before the data analysis [5].

Qvalue=pvalue×total sample number/samplerank

### 2.7 Real‐Time Quantitative Polymerase Chain Reaction

Total RNA was isolated from HepG2 cells (ATCC, Manassas, VA, USA) using the RNeasy Mini Kit (Qiagen, Germantown, MD, USA). cDNA was synthesized using the SuperScript First‐Strand Synthesis Kit (Invitrogen, Carlsbad, CA, USA). RT‐qPCR was performed on a Prism 7900HT Sequence Detection System (Applied Biosystems, Carlsbad, CA, USA) using TaqMan assays for human PPARγ (Assay ID: Hs01115513_m1). Target gene expression levels were normalized to human GAPDH (Assay ID: Hs02786624_g1).

### 2.8 Western Blot Analysis

Western blotting was performed as previously described [7] to detect PPARγ expression. Briefly, cell lysates were homogenized in three volumes of ice‐cold 10 mM phosphate buffer (pH 7.0) containing 1 mM EDTA, 0.25 M sucrose, 1 mM sodium azide, and 0.1 mM phenylmethylsulfonyl fluoride. After centrifugation at 20,000 × g for 30 min at 4°C, protein concentrations were determined using a BCA assay (Pierce Biotechnology, Rockford, IL, USA) with bovine serum albumin as the standard. Proteins were separated by 10% SDS‐PAGE, electro‐transferred to PVDF membranes, and incubated with primary antibodies against PPARγ (CST #2443; Cell Signaling Technology, Danvers, MA, USA) and α‐Tubulin (AC012; ABclonal, Woburn, MA, USA). Protein bands were visualized using Amersham ECL Detection Reagents (Cytiva, Buckinghamshire, UK).

### 2.9 Enzyme Linked Immunosorbent Assay (ELISA) of PPARγ Protein Levels in HepG2 Cells

HepG2 cell lysates were prepared by homogenization in PBS on ice, followed by centrifugation at 5000 × g for 5 min. The supernatants were collected, and protein concentrations were measured. PPARγ protein levels in cells treated with the indicated TCM were determined using a commercial Human PPARγ ELISA Kit (Cat. No: EH3634; FineTest, Wuhan, Hubei, China) according to the manufacturer's instructions.

### 2.10 Data Statistical Analysis

XLSTAT (https://www.xlstat.com/), an add‐on of Microsoft Excel, were employed for the statistical analysis of this study. Analysis of variance (ANOVA) was applied to compare the ER difference among enhancement, ineffective, and reduction groups. Samples in the enhancement group were further divided into 10 subgroups by every tenth percentile above 10% based on their ER values and then finalizing the grouping based on the results of ANOVA followed by post hoc Bonferroni correction. The *P* value less than 0.05 was adopted as the statistically significant difference. The Z scores of the enhancement and reduction groups were compared with the corresponding ER values within each group to determine which one to be used for sample ranking within each group.

## 3 Results

### 3.1 Categorizing TCM Formulas Based on Sample P Values

A total of 139 TCM formulars were categorized into “Enhancement group”, which all demonstrated positive effects on the activity of PPARγ promoter region (*p* < 0.05), while 55 TCM formulars were categorized into “Reduction group”, which all showed negative impacts on the activity of PPARγ promoter region (*p* < 0.05). The remaining 445 formulars did not show significant impacts on the activity of PPARγ promoter region (*p* > 0.05) and, therefore, were assigned as “Ineffective group” (Table 1). The sample Q values in the enhancement group showed no significant differences (Q > 0.05) after eliminating the top one sample that had an extremely high ER value, while the sample Q values in reduction and ineffective groups were all larger than 0.05. However, the ANOVA test for all three groups showed a significant difference among the groups (*p* < 0.05). The results indicated that the categorizing method based on sample *P* values was an effective and reliable way to group tested TCM formulars.

**TABLE 1:** TCM grouping based on sample *p* values.

Round	Sample number		
	Enhancement group	Ineffective group	Reduction group
	*p* < 0.05 (ER positive)	*p* > 0.05	*p* < 0.05 (ER negative)
1	37	581	21
2	25	539	17
3	21	508	10
4	14	493	1
5	9	483	1
6	11	471	1
7	8	463	0
8	5	456	2
9	6	448	2
10	2	446	0
11	1	445	0
12	0	445	0
Subtotal	139	445	55

### 3.2 TCM Formulas That Increased the Activity of PPARγ Promoter Region

Among 139 TCM formulars with 80 SF and 59 MF in the enhancement group, the rank of sample effective rates (ER) calculated based on the experimental result was consistent with the rank of sample Z scores. Therefore, the ER values were used for sample ranking inside the group. The statistical results showed the significant differences among all the ER percentile subgroups (*p* < 0.05) except among 60%–69%, 50%–59%, and 40%–49% subgroups. However, there was a significant difference between the 60%–69% subgroup and the 40%–59% group (*p* < 0.05). Therefore, according to the statistical analysis results, all samples were eventually divided into 9 percentile subgroups (Table 2). Since there was a percentile gap between subgroups 60%–69% (S5) and 40%–59% (S6), the study would focus on the TCM formulars with their ER above 60% increase of the PPARγ promoter region activity, which included a total of 49 formulars with 14 SF over 100%, 4 SF between 90% and 99%, 12 SF and 1 MF between 80%–89%, 7 SF between 70% and 79%, 10 SF and 1 MF between 60% and 69%, respectively (Table 3).

**TABLE 2:** Subgroups of TCM formulas that increased PPARγ promoter region activity.

Subgroup	ER range	Sample number
S1	≥ 100%	14
S2	90%–99%	4
S3	80%–89%	13
S4	70%–79%	7
S5	60%–69%	11
S6	40%–59%	18
S7	30%–39%	16
S8	20%–29%	24
S9	< 20%	32
Total	—	139

**TABLE 3:** TCM formulas that increased PPARγ promoter region activity 60% and above.

Rank	Formulars ID	TCM formulars	Effective rate (%)
1	S240	*Hedysarum Polybotrys*	214
2	S241	*Astragali Radix*	152
3	S277	*Ziziphi Spinosae Semen*	136
4	S276	*Rhapontici Radix*	128
5	S270	*Talcum*	124
6	S246	*Perillae*	122
7	S237	*Achyranthis Bidentatae Radix*	112
8	S225	*Ophiopogonis Radix*	111
9	S262	*Dioscoreae Hypolaucae Rhizoma*	108
10	S247	*Violae Herba*	108
11	S263	*Cuscutae Semen*	108
12	S271	*Typhae Pollen*	106
13	S258	*Puerariae Thomsonii Radix*	100
14	S274	*Xanthii Fructus*	100
15	S226	*Asari Radix et Rhizoma*	99
16	S26	*Vitis Amurensis*	97
17	S257	*Acori Tatarinowii Rhizoma*	96
18	S224	*Hordei Germinatus Fructus*	94
19	S249	*Maydis Stigma*	89
20	S254	*Raphani Semen*	89
21	S269	*Japonica rice*	87
22	S243	*Polygonati Rhizoma*	86
23	S244	*Solanum incanum L*.	85
24	M212	Mai Wei Di Huang Wan	84
25	S265	*Angelicae Sinensis Radix*	84
26	S245	*Asteris Radix et Rhizoma*	84
27	S253	*Gynostemma Pentaphyllum Herbal seu Radix*	83
28	S273	*Atractylodis Rhizoma*	83
29	S260	*Lepidii Semen*	82
30	S267	*Drynariae Rhizoma*	81
31	S266	*Angelicae Sinensis Radix root*	80
32	S231	*Tetrapanacis Medulla*	79
33	S45	*Orthosiphon aristatus*	78
34	S233	*Gardeniae Fructus*	78
35	S223	*Epimedii Folium*	76
36	S272	*Taraxaci Herba*	76
37	S248	*Arnebiae Radix*	74
38	S234	*Houttuyniae Herba*	70
39	S261	*Allii Tuberosi Semen*	69
40	S255	*Farfarae Flos*	69
41	S228	*Ephedrae Radix et Rhizoma*	66
42	S236	*Taxilli Herba*	66
43	S229	*Citri Reticulatae Pericarpium*	66
44	S268	*Liquidamibris Fructus*	63
45	M163	Fu Ling Yin	61
46	S232	*Gardeniae Fructus*	60
47	S32	*Trogopteri Faeces*	60
48	S63	*Dictamni Cortex*	60
49	S19	*Sophorae Tonkinensis Radix et Rhizoma*	60

### 3.3 TCM Formulas That Reduced the Activity of PPARγ Promoter Region

A total of 55 TCM formulars with 40 MF and 15 SF demonstrated the reduction effects on PPARγ promoter region activity with their ER showing negative values. Among them, 23 formulars were from −91% to −99% with 6 SF and 17 MF; 8 MF and 1 SF were from −81% to −89%; 6 MF and 1 SF were from −70% to −78%; 5 MF and 4 SF were from −62% to −68%; and 4 MF and 3 SF were from −52% to −55% (Table 4).

**TABLE 4:** TCM formulas that reduced the activity of PPARγ promoter region.

Rank	Formulars ID	TCM formulars	Effective rate (%)
1	S3	*Paris polyphylla*	−99
2	S104	*Stephaniae Tetrandrae Radix*	−99
3	S345	*Gleditsia sinensis*	−99
4	M269	Ning Sou Hua Tan Tang	−99
5	M89	Ping Wei San	−99
6	M87	Ban Xia Xie Xin Tang	−99
7	M85	Bai Tou Weng Tang	−98
8	M86	Ban Xia Hou Po Tang	−98
9	M88	Ban Xia Bai Shu Tian Ma Tang	−98
10	M94	Sheng Jiang Xie Xin Tang	−98
11	M93	Sheng Hua Tang	−98
12	M147	Xiang Ru Yin	−98
13	S169	*Gymnema sylvestre leaf*	−98
14	S31	*Chinensis Galla*	−97
15	M90	Yu Quan Wan	−97
16	M140	Chai Hu Gui Zhi Qian Jiang Tang	−96
17	M83	Bai Hu Tang	−95
18	M268	Ning Sou Wan	−95
19	M132	Di Dang Tang	−93
20	M95	Sheng Mai Yin	−92
21	S340	*Paris polyphylla*	−92
22	M91	Yu Nv Jian	−91
23	M261	Dun Sou San	−91
24	M84	Bai Hu Jia Ren Shen Tang	−89
25	M190	Qing Xin Li Ge Tang	−88
26	M148	Wei Ling Tang	−87
27	M92	Yu Ping Feng San	−86
28	M270	Tiao Wei Cheng Qi Tang	−84
29	M127	Ding Chuan Tang	−84
30	M105	Fang Feng Tong Sheng San	−81
31	M113	Shao Yao Gan Cao Tang	−81
32	S90	*Albiziae Flos*	−81
33	M61	Mu Xiang Bin Lang Wan	−78
34	M96	Zuo Gui Wan	−78
35	M180	Shen Mi Tang	−77
36	M177	Tao Ren Cheng Qi Tang	−76
37	S71	*Granati Pericarpium*	−74
38	M288	Xie Bai San	−74
39	M201	Ma Huang Tang	−70
40	S191	*Mori Folium*	−68
41	S192	*Mori Cortex*	−68
42	M229	Yue Bi Jia Shu Tang	−67
43	S190	*Mori Ramulus*	−66
44	M101	Zhu Ru Wen Dan Tang	−64
45	M133	Zhi Suo Er Chen Tang	−63
46	M5	Ren Shen Xie Fei Tang	−62
47	S4	*Caryophylli Flos*	−62
48	M202	Ma Xing Gan Shi Tang	−62
49	S140	*Acacia catechu*	−55
50	M203	Ma Zi Ren Wan	−54
51	S96	*Paeoniae Rubra Radix*	−53
52	M224	Huang Qin Tang	−52
53	M68	Zhi Sou San	−52
54	S112	*Alpiniae Officinarum Rhizoma*	−52
55	M135	Chai Ge Jie Ji Tang	−52

### 3.4 Validation of TCM Formula Efficacy in the HepG2 Cells

To evaluate the efficacy of the selected TCM formulas, we quantified and compared PPARγ gene expression, protein levels, and protein activity in HepG2 cells following treatment. Based on the screening results in Tables 3 and 4, two formulas were selected as inducer candidates—Astragali Radix (S241) and Puerariae Thomsonii Radix (S258)—while *Gymnema sylvestre* leaf (S169) and Paris polyphylla (S3) were selected as inhibitor candidates. The treatment dosage was set at 2 μg/μl, as determined by prior titration experiments (shown in S1).

**FIGURE 3:**
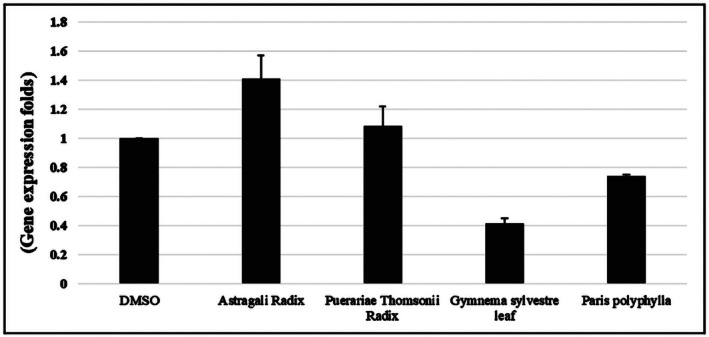
Reverse transcription‐quantitative PCR analysis of the PPARγ gene in HepG2 cells with different TCM formulas treatment. Gene expression data of PPARγ was calculated following normalization to GADPH. PPARγ, Peroxisome proliferator‐activated receptor‐gamma.

RT‐qPCR analysis revealed that PPARγ mRNA expression was significantly upregulated in HepG2 cells treated with the inducer candidates (Astragali Radix and Puerariae Thomsonii Radix) compared to the DMSO control. Conversely, PPARγ expression was markedly inhibited following treatment with the inhibitor candidates ( *Gymnema sylvestre* leaf and Paris polyphylla) (Figure 3).

**FIGURE 4:**
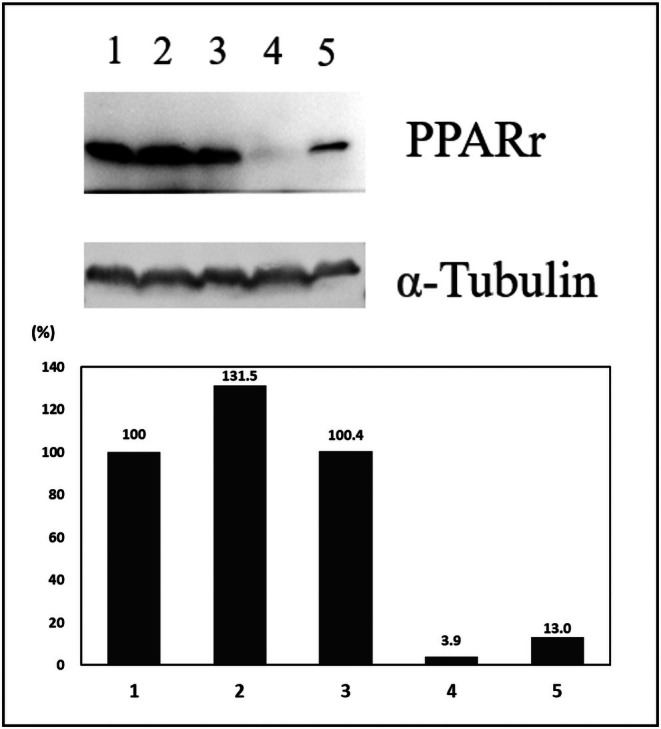
Western blot analyses of protein expression levels of PPARγ in HepG2 cells treated with (1) DMSO, (2) Astragali Radix (S241), (3) Puerariae Thomsonii Radix (S258), (4) *Gymnema sylvestre* leaf (S169), and (5) Paris polyphylla (S3). Protein expression data of PPARγ was calculated following normalization to α‐Tubulin.

Western blot analysis showed a similar trend in PPARγ protein expression levels across the different treatment groups (Figure 4). Consistent with the mRNA data, inducer and inhibitor formulas significantly differentially modulated PPARγ protein abundance in HepG2 cells.

**FIGURE 5:**
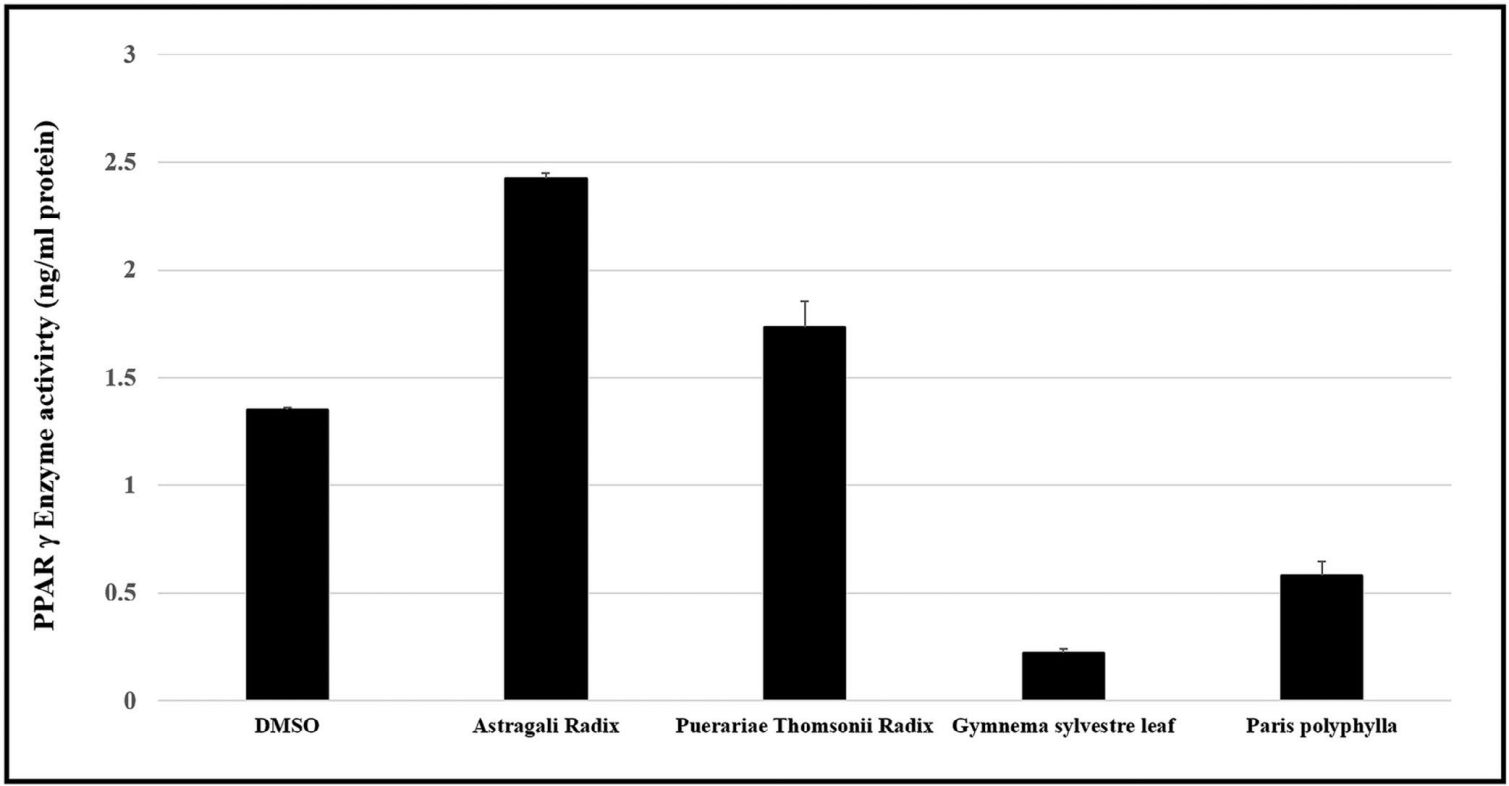
Measurement of the PPARγ protein concentrations in HepG2 cells treated with the inducer candidates (Astragali Radix and Puerariae Thomsonii Radix) and the inhibitor candidates ( *Gymnema sylvestre* leaf and Paris polyphylla) using ELISA analysis.

Furthermore, ELISA was employed to analyze PPARγ levels in HepG2 cells. The highest PPARγ protein levels were observed in cells treated with Astragali Radix (S241), whereas the lowest levels were found in the *Gymnema sylvestre* leaf (S169) group (Figure 5). Collectively, these results demonstrate that our large‐scale TCM screening platform is a robust and reliable system for drug screening and the development of novel therapeutics.

## 4 Discussion

PPARγ regulates the transcription of genes involved in glucose and lipid metabolism and improves insulin sensitivity and glucose homeostasis; therefore, it plays a critical role in T2D. The purpose of this study was to identify the potential candidate TCM formulas that demonstrated significant effects on the increasing of PPARγ promoter region activity and further might be applied in T2D treatment. The results demonstrated that 47 SF and 2 MF showed a significant increase of PPARγ promoter region activity from 60% to 214%, while 15 SF and 40 MF demonstrated a reduction of PPARγ promoter region activity from −52% to −99% compared to the positive control group after TCM treatment. The results showed that SF had a greater effect on PPARγ promoter activity than MF. In samples where PPARγ promoter activity was increased by more than 60%, SF:MF = 47:2 or 96% of SF. However, more MF demonstrated negative impacts on PPARγ promoter region activity than SF. In samples where PPARγ promoter region activity was reduced below −52%, SF:MF = 15:40 or 73% of MF, which might be due to the complexity of MF with the potential for interactions and increased toxicity among the compound single TCMs. The results suggested that TCM SF might play an important role in the discovery of novel treatment for T2D.

Among the top 49 positive impact TCM, 14 SF showed the ER over 100% with the highest one, Hedysarum Polybotrys, reaching 214%. The results were consistent with the previous studies that Hedysarum Polybotrys [8], Astragali Radix [9], Ziziphi Spinosae Semen [10], Perillae [11], Ophiopogonis Radix [12], Dioscoreae Hypolaucae Rhizoma [13], Cuscutae Semen [14], Typhae Pollen [15], Puerariae Thomsonii Radix [16], Xanthii Fructus [17] could improve insulin secretion, reduce insulin resistance, regulate substance metabolism, regulate gut microbiota, and reduce blood sugar levels, which demonstrated a good therapeutic effect on T2D. There were no direct connections found between Rhapontici Radix, Talcum, Achyranthis Bidentatae Radix, and Violae Herba and management of diabetes. However, those 4 SF presented high ER to increase PPARγ promoter region activity from 108% to 128% and would be considered potential candidates for diabetes management. Except Hordei Germinatus Fructus that could help lower blood glucose levels and improve insulin sensitivity [18], Asari Radix et Rhizoma, *Vitis Amurensis* , Acori Tatarinowii Rhizoma had no solid research evidence to directly link them to diabetes management even the results of this study showing the high ER of promoting PPARγ promoter region activity from 94% to 99% comparing to the control group. Further studies are therefore warranted on those 3 SF. In the group with ER from 80% to 89%, Maydis Stigma [19], Polygonati Rhizoma [20], *Solanum incanum* L [21], Gynostemma Pentaphyllum Herbal seu Radix [22], Atractylodis Rhizoma [23] have been reported to lower blood glucose levels and improve insulin sensitivity, while Raphani Semen may have beneficial effects on hypertension, obesity, and diabetes mellitus [24]. Angelicae Sinensis Radix, Asteris Radix et Rhizoma, Lepidii Semen, Drynariae Rhizoma, and Angelicae Sinensis Radix root had no previous report about their links to diabetes management, but in this study, they all demonstrated an ER to increase PPARγ promoter region activity from 80% to 84% compared to the control group, indicating their potential benefits in diabetes management. Japonica rice has a high glycemic index (GI) and glycemic load (GL) for a rapid increase in blood sugar levels and should be considered a risk factor for diabetes. However, this study showed that Japonica rice could effectively increase the PPARγ promoter region activity by 87% compared to the control group, which brought a new view to this SF, and further investigation was needed. The MF, Mai Wei Di Huang Wan, which contains Rehmannia glutinosa, *Cornus officinalis* , *Alisma plantago‐aquatica* , Atractylodes macrocephala, and *Paeonia lactiflora* , showed an ER of 84% compared with the control group. The results confirmed the potential benefit of this MF in diabetes management, but further studies are necessary to identify the active ingredients of this MF. Orthosiphon aristatus [25], Gardeniae Fructus (Water) [26], Epimedii Folium [27], Taraxaci Herba [28], and Houttuyniae Herba [29], Gardeniae Fructus (Mountain) [26], Taxilli Herba [30], Citri Reticulatae Pericarpium [31] have been reported to help lower blood glucose levels and improve insulin sensitivity, while Tetrapanacis Medulla, Arnebiae Radix, Allii Tuberosi Semen, Farfarae Flos, Ephedrae Radix et Rhizoma, Liquidamibris Fructus, Trogopteri Faeces, Dictamni Cortex, Sophorae Tonkinensis Radix et Rhizoma, and Fu Ling Yin had no report directly relating to diabetes management although they all showed an ER between 60%–79% in this study.

Among 55 formulars in the reduction group, 13 formulars including 7 MF of Ban Xia Xie Xin Tang [32], Sheng Jiang Xie Xin Tang [33], Sheng Mai Yin [34], Bai Hu Jia Ren Shen Tang [35], Fang Feng Tong Sheng San [36], Zuo Gui Wan [37], Zhu Ru Wen Dan Tang [38] and 6 SF of *Gymnema Sylvestre* leaf [39], Granati Pericarpium [40], Mori Folium [41], Mori Cortex [42], Mori Ramulus [43], Paeoniae Rubra Radix [44] have been reported in past studies with the effects to reduce blood glucose levels, improve insulin resistance, improve lipid metabolism, and anti‐inflammation; therefore, they can be used in blood sugar management and potential diabetic treatment. In addition, all the rest formulars are considered to have potentials of anti‐inflammatory effect, antioxidant activity, and hypoglycemic effect based on the theories of TCM, which makes them all potential candidates for blood sugar and diabetic side effects management. However, the results of this study demonstrated that all 13 reported formulars that may help in diabetic management showed the reduction of PPARγ promoter region activity from −52% to −99%. Moreover, all 55 formulars in the reduction group at least reduced PPARγ promoter region activity 50% and above, which would significantly reduce the expression of PPARγ gene and subsequently affect glucose and lipid metabolism in body tissues by reducing the expression of GLUT4 to reduce cell insulin sensitivity, reducing adiponectin to lower anti‐inflammatory effects, reducing FABPs and fatty acid translocase CD36 to directly affect lipid transportation, and LPL to reduce the breakdown of triglyceride. All of those would affect body cell adipogenesis and insulin sensitivity, and therefore, the blood glucose level. The results of this study oppose the recommendations of those 55 TCM formulars to be used in the management of blood glucose and alleviate diabetic side effect symptoms. Although they all possess anti‐inflammatory and antioxidant effects and may be useful in certain cases in clinical practice, for the long‐term consideration of diabetic management, their potential effects on the reduction of PPARγ promoter region activity should be considered for comprehensive evaluation on each specific case to balance the clinical benefit and long‐term diabetic management.

The identification of negative regulators in this study, such as *Gymnema sylvestre* (S169), warrants further physiological interpretation. Traditionally, PPARγ agonism has been the primary therapeutic strategy for T2DM; however, full activation is often associated with adverse effects like weight gain and adipocyte hypertrophy. We posit that the negative regulators identified herein may not function as simple antagonists, but rather as selective PPARγ modulators (SPPARMs). According to the paradigm established by Choi et al. [45], certain compounds can improve insulin resistance by blocking the pathological phosphorylation of PPARγ at Ser273—a modification mediated by CDK5 in obese states—rather than through classical transcriptional agonism. By interfering with such posttranslational modifications or fine‐tuning the recruitment of specific coregulators, these TCM formulas might restore the expression of insulin‐sensitizing genes (e.g., Adiponectin) without triggering excessive adipogenesis. This provides a sophisticated molecular basis for the “bidirectional regulation” or homeostatic effects often observed in Traditional Chinese Medicine (TCM) when managing complex metabolic disorders.

## 5 Conclusion

This study explored a large group of TCM including SF and MF to exam the drug effective rate on PPARγ promoter region activity and therefore potentially improve insulin sensitivity and regulate blood glucose level. Among 639 TCM formulas, 139 formulas (22%) demonstrated positive impacts with 49 formulas (8%) showing the effectiveness of increasing PPARγ promoter region activity by over 60%, while 55 formulas (9%) showed the negative impacts on PPARγ promoter region activity by below −52%. 445 formulas (70%) showed no significant effect. Among the enhancement group, 96% formulas with ER increase over 60% were SF, while in the reduction group, 73% formulas were MF. A total of 49 formulas which included 24 reported SF and newly discovered 23 SF and 2 MF increased PPARγ promoter region activity by over 60%, therefore, potentially could be the candidates for T2D management. Future studies on the mechanisms of those potential diabetic management candidates are warranted. Moreover, 55 formulas were confirmed to negatively impact the PPARγ promoter region activity; therefore, although they all showed the potential benefits for alleviation of diabetic side effects, a comprehensive evaluation based on the clinical benefit and long‐term diabetic management should be made. In addition, the biostatistical data processing strategy of this study has been confirmed effective and accurate in large amount drug screening, which can be applied in other pharmaceutical and pharmacological research fields.

## Funding

This work was supported by “Oklahoma Louis Stokes Alliance for Minority Participation” (OK‐LSAMP) funded by the National Science Foundation, “Organized Research Grant” funded by Southeastern Oklahoma State University (290‐01‐121‐0502‐00), and China Medical University Hospital in Taiwan (grant nos. DMR‐113‐104).

## Conflicts of Interest

The authors declare no conflicts of interest.

## Supporting information


**Figure S1:** Cytotoxicity assay in HepG2 cells treated with TCM formula (M215).

## Data Availability

The data that support the findings of this study are available from the corresponding author upon reasonable request. The Supporting Information is available in the supplementary section.

## References

[1] K. C. Chen , S. S. Chang , H. J. Huang , T. L. Lin , Y. J. Wu , and C. Y. C. Chen , “Three‐In‐One Agonists for PPAR‐α, PPARγ, and PPAR‐δ From Traditional Chinese Medicine,” Journal of Biomolecular Structure & Dynamics 30 (2012): 662–683. 22731403 10.1080/07391102.2012.689699

[2] T. Chen , Y. Zhang , Y. Liu , et al., “MiR‐27a Promotes Insulin Resistance and Mediates Glucose Metabolism by Targeting PPARγ‐Mediated PI3K/AKT Signaling,” Aging (Albany NY) 11 (2019): 7510–7524. 31562809 10.18632/aging.102263 PMC6781997

[3] F. Mohammadpour , H. Darmani‐Kuhi , A. Mohit , and M. M. Sohani , “Obesity, Insulin Resistance, Adiponectin, and PPARγ Gene Expression in Broiler Chicks Fed Diets Supplemented With Fat and Green Tea ( *Camellia sinensis* ) Extract,” Domestic Animal Endocrinology 72 (2020): 106440. 32247991 10.1016/j.domaniend.2020.106440

[4] L. Maréchal , M. Laviolette , A. Rodrigue‐Way , et al., “The CD36‐PPARγ Pathway in Metabolic Disorders,” International Journal of Molecular Sciences 19 (2018): 1529. 29883404 10.3390/ijms19051529 PMC5983591

[5] M. Laplante , H. Sell , K. L. MacNaul , D. Richard , J. P. Berger , and Y. Deshaies , “PPAR‐Gamma Activation Mediates Adipose Depot‐Specific Effects on Gene Expression and Lipoprotein Lipase Activity: Mechanisms for Modulation of Postprandial Lipemia and Differential Adipose Accretion,” Diabetes 52 (2003): 291–299. 12540599 10.2337/diabetes.52.2.291

[6] J. M. Lenhard , “PPAR Gamma/RXR as a Molecular Target for Diabetes,” Receptors and Channels 7 (2001): 249–258. 11697231 10.1111/j.1651-2227.2001.tb03250.x

[7] Y. H. Tang , Y. H. Wang , C. C. Chen , C. J. Chan , F. J. Tsai , and S. Y. Chen , “Genetic and Functional Effects of Adiponectin in Type 2 Diabetes Mellitus Development,” International Journal of Molecular Sciences 23 (2022): 13544. 36362336 10.3390/ijms232113544 PMC9658884

[8] F. Hu , X. Li , L. Zhao , et al., “C. Antidiabetic Properties of Purified Polysaccharide From Hedysarum Polybotrys,” Canadian Journal of Physiology and Pharmacology 88 (2010): 64–72. 20130740 10.1139/Y09-098

[9] C. Li , K. Zhang , L. Liu , et al., “Study of the Mechanism of Astragali Radix in Treating Type 2 Diabetes Mellitus and Its Renal Protection Based on Enzyme Activity, Network Pharmacology, and Experimental Verification,” Molecules 28 (2023): 8030. 38138520 10.3390/molecules28248030 PMC10745890

[10] M. H. Liu , H. X. Jin , Z. Song , et al., “Phytochemical, Pharmacological, Pharmacokinetic and Toxicological Characteristics of Ziziphi Spinosae Semen: A Review,” Frontiers in Pharmacology 15 (2024): 1504009. 39679366 10.3389/fphar.2024.1504009 PMC11639084

[11] A. Dhyani , R. Chopra , and M. Garg , “A Review on Nutritional Value, Functional Properties and Pharmacological Application of Perilla ( *Perilla frutescens* L.),” Biomedical and Pharmacology Journal 12 (2019): 649–660.

[12] L. Ding , P. Li , C. B. Lau , et al., “Mechanistic Studies on the Antidiabetic Activity of a Polysaccharide‐Rich Extract of Radix Ophiopogonis,” Phytotherapy Research 26, no. 1 (2012): 101–105. 21560174 10.1002/ptr.3505

[13] L. Sun , Y. M. Di , C. Lu , et al., “Additional Benefit of Chinese Medicine Formulae Including Dioscoreae Rhizome (Shanyao) for Diabetes Mellitus: Current State of Evidence,” Frontiers in Endocrinology (Lausanne) 11 (2020): 553288. 10.3389/fendo.2020.553288 PMC7685178 33244311

[14] M. Rahmatullah , S. Sultan , T. T. Toma , et al., “Effect of *Cuscuta reflexa* Stem and *Calotropis procera* Leaf Extracts on Glucose Tolerance in Glucose‐Induced Hyperglycemic Rats and Mice,” African Journal of Traditional, Complementary, and Alternative Medicines 7 (2009): 109–112. 10.4314/ajtcam.v7i2.50864 PMC3021163 21304621

[15] X. T. Feng , Q. Chen , Z. Xie , et al., “Pollen Typhae Total Flavone Improves Insulin Resistance in High‐Fat Diet and Low‐Dose Streptozotocin‐Induced Type 2 Diabetic Rats,” Bioscience, Biotechnology, and Biochemistry 78 (2014): 1738–1742. 25273139 10.1080/09168451.2014.930318

[16] J. Li , H. Zhang , H. Ouyang , et al., “Pueraria Thomsonii Radix Water Extract Alleviate Type 2 Diabetes Mellitus in Db/Db Mice Through Comprehensive Regulation of Metabolism and Gut Microbiota,” Molecules 28 (2023): 7471. 38005193 10.3390/molecules28227471 PMC10673130

[17] X. Li , Z. Li , M. Xue , et al., “Fructus Xanthii Attenuates Hepatic Steatosis in Rats Fed on High‐Fat Diet,” PLoS One 8 (2013): e61499. 23585904 10.1371/journal.pone.0061499 PMC3621865

[18] H. Wei , X. Liang , B. Wu , et al., “Antihyperglycemic and Antioxidant Activity of Fructus Hordei Germinatus Extract on Streptozotocin‐Induced Diabetic Rats,” Tropical Journal of Pharmaceutical Research 14 (2015): 1651–1657.

[19] L. Sheng , Q. Chen , L. Di , and N. Li , “Evaluation of Anti‐Diabetic Potential of Corn Silk in High‐Fat Diet/Streptozotocin‐Induced Type 2 Diabetes Mice Model,” Endocrine, Metabolic & Immune Disorders Drug Targets 21 (2021): 131–138. 10.2174/1871530320666200606224708 32504506

[20] A. Kato and T. Miura , “Hypoglycemic Activity of Polygonati Rhizoma in Normal and Diabetic Mice,” Biological & Pharmaceutical Bulletin 16 (1993): 1118–1120. 8312868 10.1248/bpb.16.1118

[21] S. Sabiu , E. O. Ajani , R. A. Aladodo , et al., “Membrane Stabilization and Probable Mechanisms of Hypoglycemic Activity of Fruit Extract of *Solanum incanum* L. (Solanaceae),” Comparative Clinical Pathology 27 (2018): 1611–1619.

[22] V. T. T. Huyen , D. V. Phan , P. Thang , et al., “Gynostemma Pentaphyllum Tea Improves Insulin Sensitivity in Type 2 Diabetic Patients,” Journal of Nutrition and Metabolism 2013 (2013): 765383. 23431428 10.1155/2013/765383 PMC3572697

[23] Y. Han , H. W. Jung , and Y. K. Park , “The Roots of Atractylodes Japonica Koidzumi Promote Adipogenic Differentiation via Activation of the Insulin Signaling Pathway in 3T3‐L1 Cells,” BMC Complementary and Alternative Medicine 12 (2012): 154. 22978376 10.1186/1472-6882-12-154 PMC3552989

[24] L. Gao , H. Li , B. Li , et al., “Traditional Uses, Phytochemistry, Transformation of Ingredients and Pharmacology of the Dried Seeds of *Raphanus sativus* L. (Raphani Semen), a Comprehensive Review,” Journal of Ethnopharmacology 294 (2022): 115387. 35580770 10.1016/j.jep.2022.115387

[25] F. I. Abdullah , L. S. Chua , S. P. M. Bohari , and E. Sari , “Rationale of Orthosiphon Aristatus for Healing Diabetic Foot Ulcer,” Natural Product Communications 15 (2020): 1–13.

[26] Q. Yu , T. Takahashi , M. Nomura , and S. Kobayashi , “Anti‐Hyperglycemic Effect of Single Administered Gardeniae Fructus in Streptozotocin‐Induced Diabetic Mice by Improving Insulin Resistance and Enhancing Glucose Uptake in Skeletal Muscle,” Chinese Medicine 4 (2013): 157–165.

[27] R. Huang , L. Xu , T. Chen , et al., “Epimedii Folium Polysaccharide Ameliorated Glucose Metabolic Disorder in Type 2 Diabetic Mice by Regulating the SIRT1/PPARγ Signaling Pathway,” Indian Journal of Pharmaceutical Education And Research 56 (2022): s274–s280.

[28] M. Kania‐Dobrowolska and J. Baraniak , “Dandelion ( *Taraxacum officinale* L.) as a Source of Biologically Active Compounds Supporting the Therapy of Co‐Existing Diseases in Metabolic Syndrome,” Food 11 (2022): 2858. 10.3390/foods11182858 PMC9498421 36140985

[29] S. C. Shao and H. Y. Wang , “Therapeutic Effects of Compound Herba Houttuyniae in Type 2 Diabetic Rats,” Chinese Medical Journal 133 (2020): 877–878. 32187059 10.1097/CM9.0000000000000701 PMC7147645

[30] M. Qin , Q. Huang , X. Yang , et al., “Taxillus Chinensis (DC.) Danser: A Comprehensive Review on Botany, Traditional Uses, Phytochemistry, Pharmacology, and Toxicology,” Chinese Medicine 17 (2022): 136. 36482376 10.1186/s13020-022-00694-5 PMC9730624

[31] Y. Wang , X. Zhang , C. Zhou , H. Khan , M. Fu , and W. S. Cheang , “Citri Reticulatae Pericarpium (Chenpi) Protects Against Endothelial Dysfunction and Vascular Inflammation in Diabetic Rats,” Nutrients 14 (2022): 5221. 36558380 10.3390/nu14245221 PMC9783663

[32] M. Yang , Z. Hu , L. Zhang , and R. Yue , “Effects and Mechanisms of Ban‐Xia Xie‐Xin Decoction on Type 2 Diabetes Mellitus: Network Pharmacology Analysis and Experimental Evidence,” Endocrine, Metabolic & Immune Disorders Drug Targets 23 (2023): 947–963. 10.2174/1871530323666221220141716 36545745

[33] O. Djakpo and W. Yao , “ *Rhus chinensis* and Galla Chinensis—Folklore to Modern Evidence: Review,” Phytotherapy Research 24 (2010): 1739–1747. 20564459 10.1002/ptr.3215 PMC7167973

[34] M. Yang , Z. Hu , and R. Yue , “Effects of Sheng‐Mai Injection on Diabetes Mellitus: A Systematic Review and Meta‐Analysis,” Endocrine, Metabolic & Immune Disorders Drug Targets 8 (2023): 1051–1067. 10.2174/1871530323666230127121738 36705242

[35] L. Wang , X. Shen , F. Wang , and X. Xu , “UHPLC–Q/Orbitrap/MS/MS Fingerprinting of Bai‐Hu‐Jia‐Ren‐Shen‐Tang Decoction and Evaluation of Its Antioxidant Activity in Streptozotocin‐Induced Diabetic Rats,” Acta Chromatographica 35 (2023): 293–301.

[36] S. Gao , H. Satsu , and T. Makino , “Inhibitory Effect of Bofutsushosan (Fang Feng Tong Sheng San) on Glucose Transporter 5 Function In Vitro,” Journal of Natural Medicines 72 (2018): 530–536. 29423591 10.1007/s11418-018-1183-0

[37] Y. X. Z. Xu , S. Xi , and X. Qian , “Evaluating Traditional Chinese Medicine and Herbal Products for the Treatment of Gestational Diabetes Mellitus,” Journal of Diabetes Research 2019 (2019): 9182595. 31886289 10.1155/2019/9182595 PMC6915007

[38] M. Alkhatib , C. Fayad , A. Badran , et al., “Preventive and Therapeutic Effects of *Punica granatum* (Pomegranate) in Respiratory and Digestive Diseases: A Review,” Applied Sciences 12 (2022): 12326.

[39] P. Kanetkar , R. Singhal , and M. Kamat , “ *Gymnema sylvestre* : A Memoir,” Journal of Clinical Biochemistry and Nutrition 41 (2007): 77–81. 18193099 10.3164/jcbn.2007010 PMC2170951

[40] Ö. Özpak Akkuş , U. Metin , and Z. Çamlık , “The Effects of Pomegranate Peel Added Bread on Anthropometric Measurements, Metabolic and Oxidative Parameters in Individuals With Type 2 Diabetes: A Double‐Blind, Randomized, Placebo‐Controlled Study,” Nutrition Research and Practice 17 (2023): 698–716. 37529273 10.4162/nrp.2023.17.4.698 PMC10375327

[41] K. Lyu , W. Yue , J. Ran , Y. Liu , and X. Zhu , “In Vivo Therapeutic Exploring for Mori Folium Extract Against Type 2 Diabetes Mellitus in Rats,” Bioscience Reports 41 (2021): BSR20210977. 34724560 10.1042/BSR20210977 PMC8661501

[42] L. Ma , H. Ni , X. Zou , et al., “Mori Cortex Prevents Kidney Damage Through Inhibiting Expression of Inflammatory Factors in the Glomerulus in Streptozocin‐Induced Diabetic Rats,” Iranian Journal of Basic Medical Sciences 20 (2017): 715–721. 28868127 10.22038/IJBMS.2017.8842 PMC5569450

[43] T. Han , E. Ko , M. Kim , et al., “Mori Ramulus Inhibits Pancreatic β‐Cell Apoptosis and Prevents Insulin Resistance by Restoring Hepatic Mitochondrial Function,” Antioxidants (Basel) 10 (2021): 901. 34204891 10.3390/antiox10060901 PMC8229938

[44] C. C. Chang , W. Yuan , Y. L. Lin , et al., “Evaluation of the In Vivo Therapeutic Effects of Radix Paeoniae Rubra Ethanol Extract With the Hypoglycemic Activities Measured From Multiple Cell‐Based Assays,” Evidence‐Based Complementary and Alternative Medicine 2016 (2016): 32627906. 10.1155/2016/3262790 PMC5153506 28018473

[45] J. H. Choi , A. S. Banks , J. L. Estall , et al., “Anti‐Diabetic Drugs Inhibit Obesity‐Linked Phosphorylation of PPARgamma by Cdk5,” Nature 466 (2010): 451–456. 20651683 10.1038/nature09291 PMC2987584

