# Supplementary material for: Large‐Scale Evaluation of Traditional Chinese Medicines Reveals Potential PPARγ Modulators for Type 2 Diabetes Management
Source: J Clin Lab Anal. 2026 Apr 1;40(12):e70215. doi: 10.1002/jcla.70215 (PMC13327478; doi:10.1002/jcla.70215)
Supplement: Supplementary file 1 — Figure S1: Cytotoxicity assay in HepG2 cells treated with TCM formula (M215). [file JCLA-40-e70215-s001.docx]

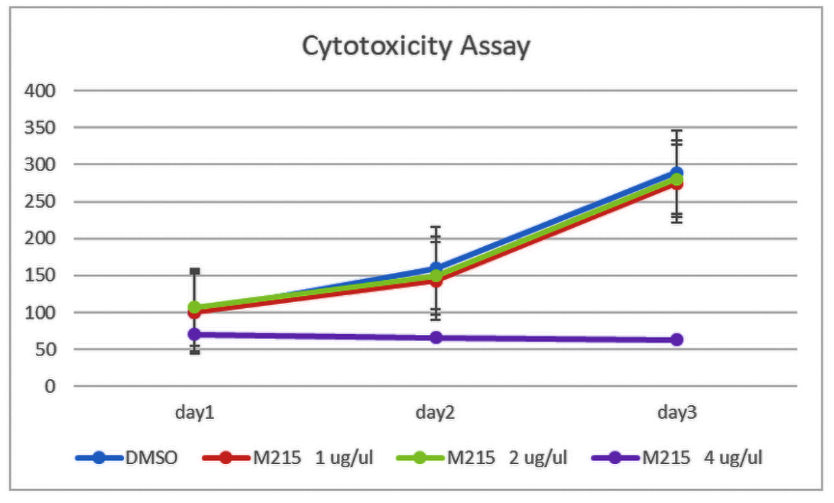


|  | day1 | | day2 | | day3 | |
| --- | --- | --- | --- | --- | --- | --- |
|  | Mean | SD | Mean | SD | Mean | SD |
| DMSO | 100.00 | 6.84 | 159.93 | 7.42 | 289.45 | 13.03 |
| M215 1 ug/ul | 100.10 | 1.56 | 142.69 | 14.76 | 274.61 | 22.46 |
| M215 2 ug/ul | 106.55 | 7.16 | 149.61 | 8.45 | 280.42 | 10.99 |
| M215 4 ug/ul | 70.25 | 13.01 | 65.66 | 13.77 | 63.21 | 13.27 |

**Supplemental Figure S1.** Cytotoxicity assay in HepG2 cells treated with TCM formula (M215).
